# Association of running manner with bacterial community dynamics in a partial short-term nitrifying bioreactor for treatment of piggery wastewater with high ammonia content

**DOI:** 10.1186/s13568-016-0245-5

**Published:** 2016-09-15

**Authors:** Wei-Li Du, Qiang Huang, Li-Li Miao, Ying Liu, Zhi-Pei Liu

**Affiliations:** 1 State Key Laboratory of Microbial Resources, Institute of Microbiology, Chinese Academy of Sciences, No. 1 West Beichen Road, Chaoyang District, Beijing, 100101 People’s Republic of China; 2 University of Chinese Academy of Sciences, Beijing, 100049 People’s Republic of China

**Keywords:** Partial short-term nitrifying bioreactor, Sequencing batch manner (SBM), Bacterial community dynamics, Ammonia-oxidizing bacteria (AOB), Piggery wastewater

## Abstract

Optimization of running parameters in a bioreactor requires detailed understanding of microbial community dynamics during the startup and running periods. Using a novel piggery wastewater treatment system termed “UASB + SHARON + ANAMMOX” constructed in our laboratory, we investigated microbial community dynamics using the Illumina MiSeq method, taking activated sludge samples at ~2-week intervals during a ~300-day period. Ammonia-oxidizing bacteria (AOB) were further investigated by quantification of AOB *amoA* genes and construction of gene clone libraries. Major changes in bacterial community composition and dynamics occurred when running manner was changed from continuous flow manner (CFM) to sequencing batch manner (SBM), and when effluent from an upflow anaerobic sludge blanket (UASB) reactor for practical treatment of real piggery wastewater was used as influent; differences among these three experimental groups were significant (*R*^*2*^ = 0.94, *p* < 0.01). When running manner was changed from CFM to SBM, relative abundance of the genus *Nitrospira* decreased sharply from 18.1 % on day 116 to 1.5 % on day 130, and to undetectable level thereafter. Relative abundance of the genus *Nitrosomonas* increased from ~0.67 % during the CFM period to 8.0 % by day 220, and thereafter decreased to a near-constant ~1.6 %. Environmental factors such as load ammonia, effluent ammonia, effluent nitrite, UASB effluent, pH, and DO levels collectively drove bacterial community dynamics and contributed to maintenance of effluent NH_4_^+^-N/NO_2_^−^-N ratio ~1. Theses results might provide useful clues for the control of the startup processes and maintaining high efficiency of such bioreactors.

**Electronic supplementary material:**

The online version of this article (doi:10.1186/s13568-016-0245-5) contains supplementary material, which is available to authorized users.

## Introduction

Ammonia, a common aquatic pollutant, is a cause of numerous environmental problems. Wastewater from piggeries (pig farms) contains high levels of chemical oxygen demand (COD) and ammonia (Bernet et al. 2000; Zhu et al. 2013) and is a major source of ammonia pollution (Bernet et al. 1996; Li et al. 2012). Anaerobic digestion coupled with biogas production, as utilized in upflow anaerobic sludge blanket (UASB) reactors, is a widely used and effective method of COD removal (Hashimoto 1983; Llabrés-Luengo and Mata-Alvarez 1987; Lo et al. 1994). On the other hand, nitrogen removal methods generally rely on the conventional nitrification–denitrification process, which consumes huge amounts of oxygen and organic matter (Bernet et al. 1996; Boiran et al. 1996; Odegaard 1988).

Anaerobic ammonium oxidation (anammox/ANAMMOX) bacteria, a type of lithotrophic microorganism, were originally discovered in bioreactors of wastewater treatment plants, in which ammonium was oxidized with nitrite as electron acceptor to produce dinitrogen gas (N_2_) (Mulder et al. 1995; Strous et al. 1999; van de Graaf et al. 1995). Application of anammox bacteria for treatment of wastewater containing high ammonium and low organic matter level was shown to result in operational cost savings up to 90 % (Jetten et al. 2001). For this purpose, a single reactor system for high activity ammonium removal over nitrite (SHARON) reactor is necessary to transform ammonia into nitrite and ensure an effluent NH_4_^+^-N/NO_2_^−^-N ratio ~1 to meet the requirement of anammox bacteria (Kuenen 2008). Ammonia-oxidizing bacteria (AOB) and nitrite-oxidizing bacteria (NOB) are the main forces for aerobic oxidation of ammonia to nitrate in a SHARON reactor (Li et al. 2015). AOB, but not NOB, are expected to grow in a SHARON reactor, because nitrate accumulation is not acceptable in the process.

Many studies have focused on the structure of AOB communities in ammonia-contaminated sediment and in the activated sludge generated by treatment of ammonia-containing wastewater (Bai et al. 2012; Fitzgerald et al. 2015; Flood et al. 2015; Wang et al. 2012; Zhang et al. 2011, 2015). Concentrations of ammonia and dissolved oxygen (DO) are the key parameters that affect nitrogen removal processes as well as AOB community structure (Park and Noguera 2004). The predominant AOB species found in bioreactors is typically *Nitrosomonas europaea* (Limpiyakorn et al. 2006; Park and Noguera 2004; Wells et al. 2009), or in some cases *Nitrosomonas oligotropha* (Dionisi et al. 2002).

Depending on the running manner of bioreactors, differing parameters may strongly affect microbial community structure during adaptation to environmental changes (Turner et al. 1998; Wells et al. 2009). Microbial community composition in activated sludge directly determines the efficiency of wastewater treatment (Ibekwe et al. 2003; Wagner et al. 2002). A long period of time is necessary for generation of “seed sludge” to start a bioreactor and achieve optimal removal efficiency (Lopez et al. 2008; van der Star et al. 2007; Zheng et al. 2004). No study to date has described microbial community dynamics in activated sludge of a SHARON reactor, from the stable startup stage through wastewater treatment stage. The environmental and engineering factors that determine the dynamics of microbial community structure throughout the entire running time must be elucidated. We need to identify the relevant variables in order to design and optimize nitrification systems (Limpiyakorn et al. 2006).

We developed a novel system termed “UASB + SHARON + ANAMMOX” and evaluated its feasibility for treatment of piggery wastewater. In the present study, microbial community dynamics in a SHARON reactor were investigated using the Illumina MiSeq method, taking activated sludge samples at ~2-week intervals during a ~300-day period. AOB were further investigated by quantification of *amoA* and construction of *amoA* gene clone libraries. Our focus was the environmental and engineering factors that controlled the dynamics of microbial community succession. The AOB responsible for nitrite accumulation were evaluated throughout the running time. Our findings provided new insights into microbial community dynamics and the relationships between these dynamics and bioreactor efficiency, and will be useful in optimizing running parameters for rapid startup and stable running of partial short-term nitrifying reactors.

## Materials and methods

### SHARON reactor and running parameters

A laboratory-scale combined “UASB + SHARON + ANAMMOX” system (Additional file 1: Figure S1; Table S1) was constructed to treat piggery wastewater (COD_cr_ 600–3000 mg/L, NH_4_^+^-N 500–1500 mg/L) obtained from a husbandry base affiliated with the Chinese Academy of Agriculture Sciences, Changping District, Beijing, China. In this system, the three reactors were started up separately, and subsequently connected. This system efficiently treated piggery wastewater with effluent levels that met national discharge standards. The function of SHARON was to partially transform high-concentration ammonia to nitrite and ensure effluent NH_4_^+^-N/NO_2_^−^-N ratio ~1 to meet ANAMMOX requirements. The SHARON reactor was constructed of plexiglass [poly(methyl methacrylate)] with height 600 mm, diameter 194 mm, and effective volume 12.5 L. Activated sludge obtained from the aeration tank of a wastewater treatment plant was used as inoculum for startup. Concentrations of volatile solids and suspended solids were 3.50 and 4.87 g/L, respectively. SHARON was run at room temperature with hydraulic retention time (HRT) 25 h. The reactor was run initially in continuous flow manner (CFM) for 120 days, with DO level 0.7–1.5 mg/L, without pH control, and run subsequently in sequencing batch manner (SBM) with DO level 7.0–8.0 mg/L. The running cycle of SBM was 8 h, consisting of four stages: aerobic fill (130 min), aerobic (318 min), settle (30 min), and draw (90 s). On day 220, effluent from UASB treating real piggery wastewater was used as influent. Influent characteristics for various stages are described in Additional file 1: Table S2.

### Sample collection and DNA extraction

Activated sludge samples from SHARON were collected at ~2-week intervals during startup and running period. In total, 19 samples were obtained: 7 from the CFM period and 12 from the SBM period. Three effluent samples from UASB were also obtained.

Total DNA was extracted from each sample (~0.5 g) using a PowerSoil DNA isolation kit (MO BIO Laboratories; Shenzhen, China) according to the manufacturer’s instructions, and stored at −80 °C.

### Illumina sequencing analysis of 16S rRNA gene amplicons

Bacterial communities were analyzed for the 22 samples described above. The primer set used was 338F/806R, which targets the V3-V4 hypervariable region of bacterial 16S rRNA gene. MiSeq PE300 was used to obtain a 468-bp fragment. Raw data were processed with the Quantitative Insights Into Microbial Ecology (QIIME) toolkit, v. 1.8.0 (Caporaso et al. 2010). Chimeric sequences were checked and filtered with the UCHIME program (Caporaso et al. 2010). Quality reads were clustered into operational taxonomic units (OTUs) at 97 % sequence similarity using UPARSE (Edgar 2013). A representative sequence of each OTU was selected for taxonomic assignment using the Greengenes Database (Wang et al. 2007). For all OTU-based analyses, sequence number was normalized prior to statistical analysis by randomly resampling reads of each sample to the same size, based on the sample with the smallest sampling size. QIIME was also used to generate Bray–Curtis distance metrics (Gauch 1973) and α-diversity indices, including Chao 1 richness estimation, Shannon, ACE, Simpson, and Good’s coverage. All analyzed sequences were deposited in the National Center for Biotechnology Information (NCBI; Bethesda, MD, USA) Sequence Read Archive (SRA) database under accession number SRP072716.

### Real-time quantitative polymerase chain reaction (qPCR)

qPCR was performed on an ABI ViiA 7 quantitative thermocycler (Applied Biosystems, USA). Primer set amoA-1F/amoA-2R (Chen et al. 2008; Rotthauwe et al. 1997; Zhang et al. 2015) was used to amplify bacterial *amoA* gene. The thermal program for qPCR of bacterial *amoA* gene was: 3 min at 94 °C, 40 cycles of 30 s at 94 °C, 55 s at 60 °C, and 45 s at 72 °C (Chen et al. 2008). Primer set 341F/518R was used to quantify bacterial 16S rRNA gene, with thermal program: 3 min at 95 °C, 40 cycles of 30 s at 95 °C, 30 s at 60 °C, and 40 s at 72 °C (He et al. 2007). The 20-μL reaction mixture consisted of 10 μL 2 × KAPA SYBR FAST qPCR Master Mix^2^ Universal (KAPA Biosystems; Beijing), 0.4 μL Forward Primer (10 µM), 0.4 μL reverse primer (10 µM), 0.4 μL 50 × ROX/Low, 2 μL diluted DNA template (<20 ng), and 6.8 μL double-distilled H_2_O. A standard curve was constructed using recombined plasmid with bacterial 16S rRNA gene as template, and AOB *amoA* gene (Bai et al. 2012). All reactions, including standards and the 19 SHARON samples, were performed in triplicate.

### Bacterial *amoA* gene cloning and sequencing

Bacterial *amoA* gene libraries were constructed for 10 time points (days 1, 38, 78, 98, 116, 145, 206, 220, 235, and 261) selected during the experimental period. The qPCR products (*amoA* gene) described above were ligated to *pEASY*-T1 cloning vector, and recombined products were used to transform *Trans*1-T1 phage resistant competent cells by heat-shock method with a *pEASY*-T1 Cloning Kit (TransGen Biotech; Beijing) according to the manufacturer’s protocol. White clones were picked randomly and re-amplified using primer set M13F/M13R to screen positive clones. Screened positive colonies were subjected to sequencing. All obtained *amoA* gene sequences were deposited in the National Center for Biotechnology Information (NCBI; Bethesda, MD, USA) GenBank database under accession numbers KX215988-KX216304.

All bacterial *amoA* gene sequences obtained were clustered into different aOTUs (the term signifies differentiation from those based on 16S rRNA gene sequence) with 97 % similarity cutoff (Gao et al. 2013; Zhang et al. 2015). AOB diversity indices were calculated by QIIME as described above.

### Statistical analysis

Microbial community succession patterns in the SHARON reactor during the entire running period were determined by principle coordinate analysis (PCoA) based on Bray–Curtis distance (Gauch 1973). The ANOSIM (analysis of similarity) algorithm was used to identify notable differences among groups. Pearson’s test and redundancy analysis (RDA) were used to evaluate correlations between variable environmental factors and the dynamic microbial community. Pearson’s test was also used to evaluate correlations between variable environmental factors and major phyla or other taxonomic units of nitrifying bacteria, and between α-diversity and running parameters. The above analyses were performed with the R software program (v. 3.2.1; http://www.r-project.org). Phylogenetic trees were constructed using the MEGA 6.0 software program (Tamura et al. 2013), based on representative sequence for each OTU/aOTU, by neighbor-joining (NJ) method with bootstrap values calculated from 1000 replications.

## Results

### Performance of the SHARON reactor

**Fig. 1:**
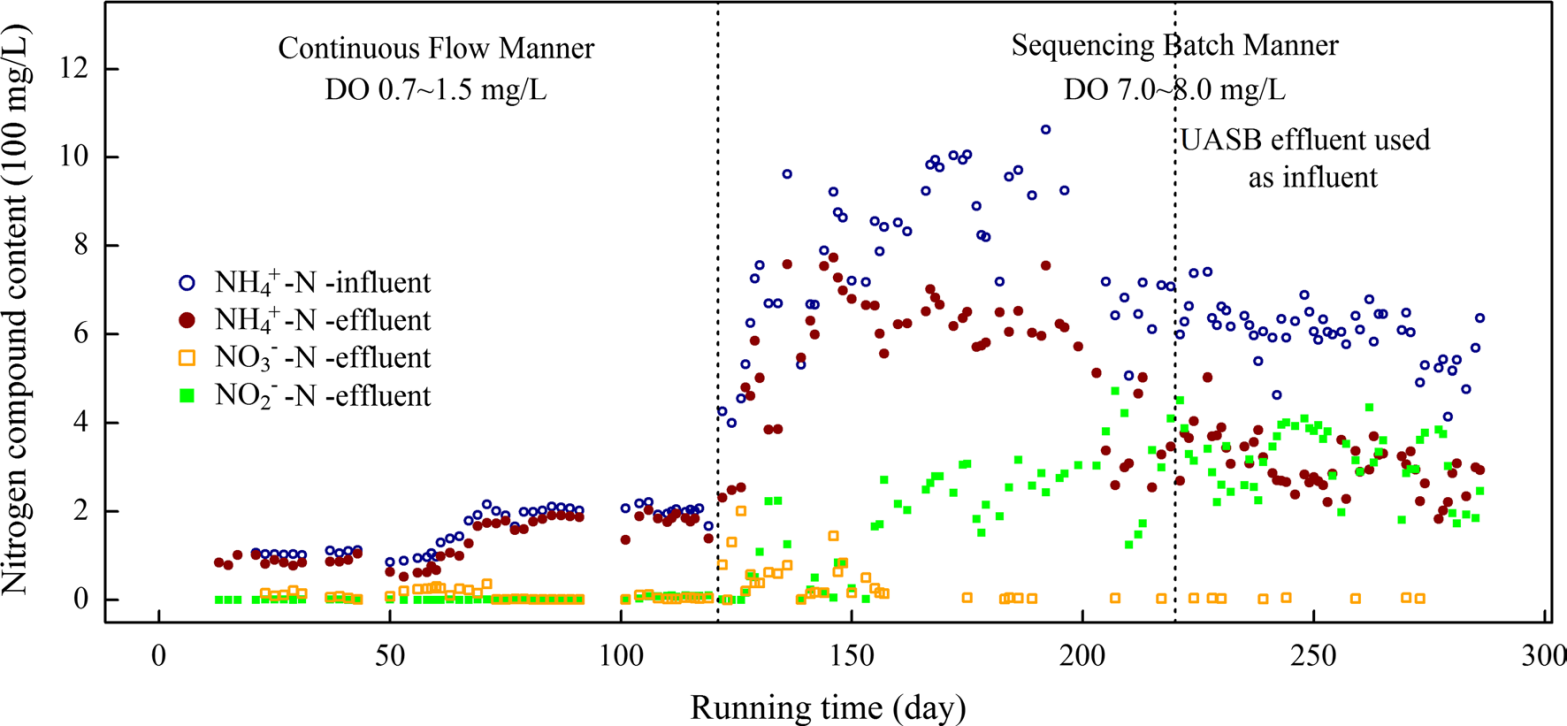
Performance of the SHARON reactor during three running stages. *Dotted lines* indicate day 121, when running manner was changed from CFM to SBM, and day 220, when effluent from UASB for practical treatment of real piggery wastewater was used as influent

The performance of the SHARON reactor was investigated during the entire experimental period, in regard to its expected ability to accumulate nitrite (but not nitrate) and to ensure effluent NH_4_^+^-N/NO_2_^−^-N ratio ~1. During the CFM period (day 1–120), with low DO level (0.7–1.5 mg/L) and low load ammonia content (NH_4_^+^-N, 100–200 mg/L) without pH control, performance was not satisfactory; i.e., the effluent contained no nitrite, low nitrate, and high ammonia (Fig. 1). This finding indicates that low DO level was not suitable for ammonia oxidation. When the running manner was changed to SBM (day 121 to end) with high DO level (7.0–8.0 mg/L) and high load ammonia content (600–900 mg NH_4_^+^-N/L, Additional file 1: Table S2) in influent with pH ~10, performance was greatly improved. Nitrite was accumulated starting on day 130, and NH_4_^+^-N/NO_2_^−^-N ratio reached ~1 on day 203, with very low nitrate content in effluent (Fig. 1), reflecting 50 % short-term nitrification (i.e., conversion of 50 % of ammonia to nitrite). When effluent from UASB-treated piggery wastewater was used as influent on day 220, NH_4_^+^-N/NO_2_^−^-N ratio was maintained at ~1 without pH adjustment, indicating the stability and efficiency of the reactor.

### Microbial community dynamics in SHARON

A total of 720,982 high-quality sequences were obtained from 851,519 total sequences of raw data following sequence processing. The size of high-quality sequences for each sample ranged from 23,554 (CFM-38) to 40,512 (CFM-98). Sequences in all samples were standardized to 23,554 for further analysis. Greengenes Database (v. 13-8) core 16S reference sequences were used for evaluation of taxonomic structure of bacterial communities, resulting in classification of 44 bacterial phyla, 116 classes, 213 orders, 337 families, and 1359 OTUs in total. Detailed phylogenetic analyses at the genus level and annotated genera are shown in Additional file 1: Figure S2.

**Fig. 2:**
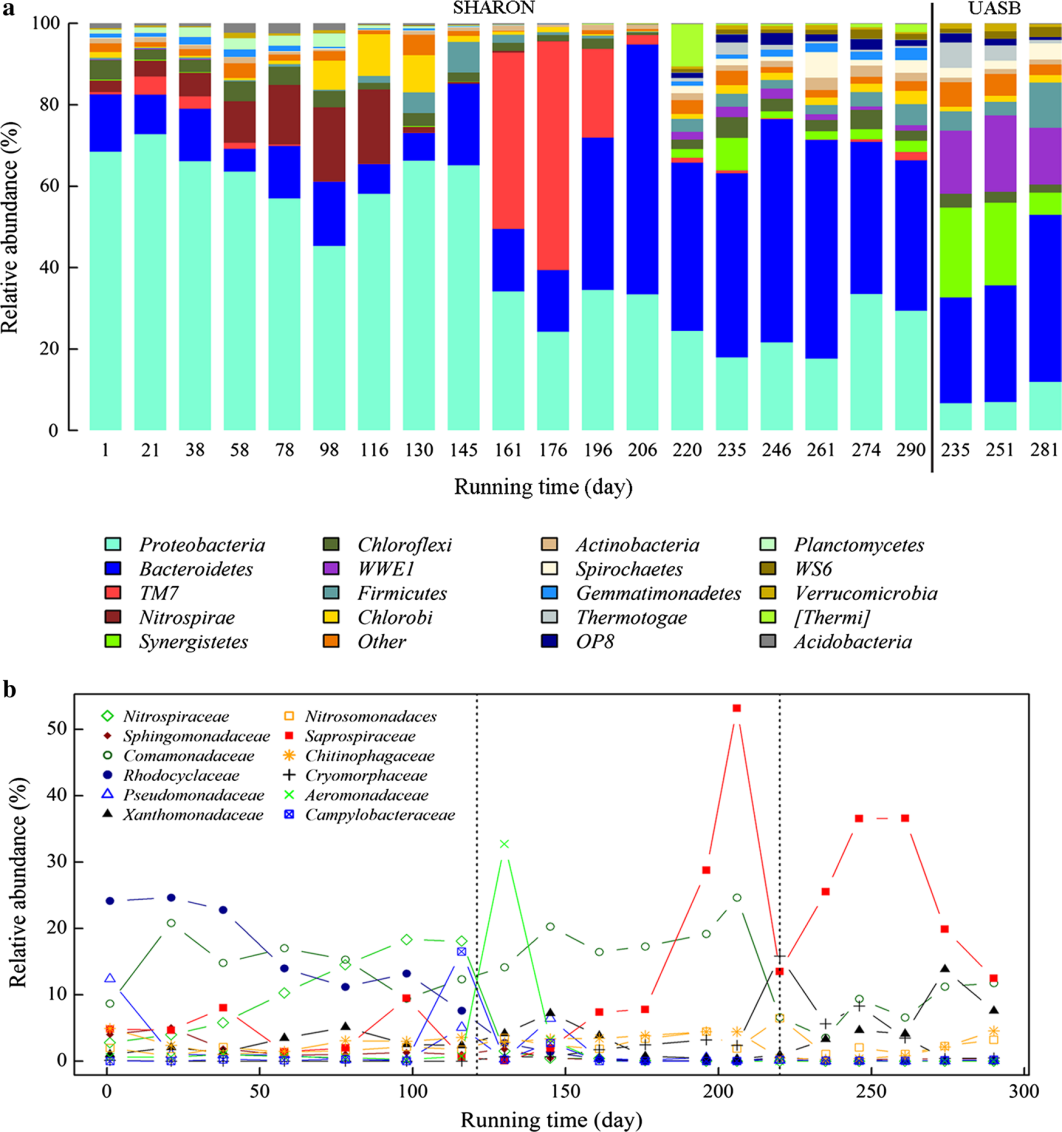
**a** Relative abundance of phyla in SHARON and UASB effluent during the entire experimental period. **b** Relative abundance of major bacterial families in SHARON

Composition of phyla in the reactor varied depending on changes in environmental and engineering factors (Fig. 2a). The two major phyla, *Proteobacteria* and *Bacteroidetes*, showed opposite trends of relative abundance during the running period (*R* = −0.79, *p* < 0.001). Relative abundance of *Proteobacteria* was in the 60–75 % range from day 1 to 145 (except for a value of 49.8 % on day 98), declined to 34.2 % on day 161, and showed little subsequent fluctuation. In striking contrast, relative abundance of *Bacteroidetes* was in the 5–20 % range from day 1 to 176, and increased to the 37–62 % range from day 177 to end of the experiment. Relative abundance of *Planctomycetes* remained ~3.3 % during the CFM period and declined to undetectable level when running manner was changed to SBM. *Nitrospira*, the major NOB, increased gradually from 2.9 % on day 1 to 18.5 % on day 116, and declined to undetectable level on day 130, 10 days after running manner was changed to SBM. Phylum *TM7* had high relative abundance (43.3 %) on day 161, decreased to 2.4 % on day 206 (when load ammonia was adjusted from 1000 to 600 mg/L), and was never higher than 2.0 % thereafter. The phyla *Spirochaetae*, *Synergistetes*, *Thermotogae*, *WWE1*, and *WS6* were detected in SHARON reactor after effluent from UASB-treated piggery wastewater was used as influent on day 220. These phyla were present in the UASB effluent, and presumably established new, stable communities in the SHARON reactor subsequently.

Relative abundances of the major bacterial families are shown in Fig. 2b. *Saprospiraceae* and *Comamonadaceae* were the predominant families in SHARON reactor. Relative abundance of *Saprospiraceae* was <10 % during days 1 to 176, increased to 53.2 % by day 206, decreased to 13.5 % by day 220, increased again to 36.6 % on day 246 and 261, and decreased gradually to 12.5 % by day 290. Relative abundance of *Comamonadaceae* decreased from 20.8 % on day 21 to 9.4 % on day 98, gradually increased to 24.6 % by day 206, fell abruptly to 6.6 % on day 220, and then increased slightly to 11.7 % by day 290. Relative abundance of *Rhodocyclaceae* decreased gradually from 24.6 to 1.4 % during the CFM period, and remained very low (~0.1 %) during the SBM period. Relative abundance of *Xanthomonadaceae* remained in the ~1.5 % range for a long time, with minor peaks of 5.1 % on day 78 and 7.2 % on day 145, increased gradually from 0.3 % on day 206 to 13.8 % on day 274, and then declined to 7.6 % on day 290. Relative abundance of *Pseudomonadaceae* remained in the 0–1.0 % range throughout the running period, except for peaks of 12.4 % on day 1, 5.1 % on day 116, and 6.4 % on day 145. Relative abundance of *Sphingomonadaceae* was 4.0 % on day 1 and 5.0 % on day 21, then declined gradually to ~1 % on day 130 and to ~0.2 % thereafter. *Chitinophagaceae* had relative abundance ~2.7 % during the entire running period. Relative abundance of *Cryomorphaceae* was low (~0.8 %) from day 1 to 206, increased to 15.8 % by day 220, then declined gradually to 0.5 % by day 290. Relative abundance of *Aeromonadaceae* was ~5 % during day 1 to 116, increased to 32.7 % by day 130, and declined to nearly undetectable level thereafter. Relative abundance of *Campylobacteraceae* was low (~0.2 %) throughout the running period, except for a peak of 16.5 % on day 116.

*Nitrospiraceae* and *Nitrosomonadaceae*, the two predominant families of autotrophic bacteria found in the SHARON reactor, play active roles in nitrogen cycling in natural environments. Relative abundance of *Nitrospiraceae*, the major NOB in SHARON, increased gradually from 2.8 % on day 1 to 18.1 % on day 116, decreased rapidly to 1.5 % by day 130, and was nearly undetectable thereafter (Fig. 2b). Relative abundance of *Nitrosomonadaceae*, the major AOB in SHARON, was in the 0.7–2.2 % range from day 1 to 116, and increased slightly to the 1.0–4.4 % range after running manner was changed to SBM, with a peak of 6.5 % on day 220.

**Fig. 3:**
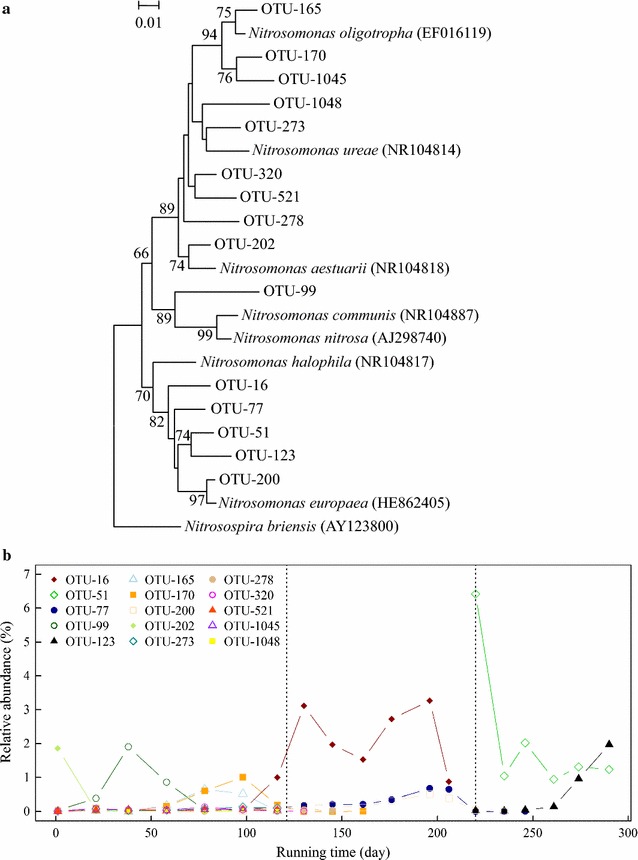
**a** Neighbor-joining tree of AOB OTUs based on 16S rRNA gene fragments. **b** Relative abundance of these OTUs during the entire experimental period. *Dotted lines* as in Fig. 1. Bootstrap values (>50 %) shown on *branch nodes* are based on 1000 trials. *Bar* evolutionary distance 0.05

Compositions of AOB communities are of great concern in regard to SHARON functioning. In total, 15 OTUs related to AOB were defined from bacterial 16S rRNA Illumina MiSeq sequences. A phylogenetic tree was constructed based on these 15 OTUs, all of which were assigned to the genus *Nitrosomonas* (Fig. 3a). Seven groups were generated from the phylogenetic tree: group 1 (OTU-165, OTU-170, OTU-1045) related to *N. oligotropha*, group 2 (OTU-273, OTU-1048) related to *N. ureae*, group 3 (OTU-320, OTU-521) related to *Nitrosomonas* spp., group 4 (OTU-278) related to *Nitrosomonas* spp., group 5 (OTU-202) related to *N. aestuarii*, group 6 (OTU-99) related to *Nitrosomonas* spp., and group 7 (OTU-16, OTU-51, OTU-77, OTU-123, OTU-200) related to *N. europaea*.

OTU-165 showed 98.4 % similarity to *N. oligotropha*. Relative abundance of OTU-165 increased gradually from 0.004 % on day 1 to 0.65 % on day 78, then declined to undetectable level on day 116 (Fig. 3b). Relative abundance of OTU-170 (97 % similarity to *N. oligotropha*) increased from 0.1 % on day 58 to 1.0 % on day 98, and subsequently declined to undetectable level. OTU-1045 (94.9 % similarity to *N. oligotropha*) was detected only during the CFM period, with relative abundance ~0.01 %. OTU-273 and OTU-1048 (96.1 and 93.9 % similarity to *N. ureae*, respectively) were detected only during the CFM period, with very low relative abundance ~0.05 %. OTU-320, OTU-521, and OTU-278 (96.1, 95.4 and 95.2 % similarity to *N. aestuarii*, respectively) were detected only during the CFM period, with low relative abundances 0.05, 0.04, and 0.05 %). Relative abundance of OTU-202 (97.5 % similarity to *N. aestuarii*) was 1.9 % on day 1 and declined to undetectable level thereafter. Relative abundance of OTU-99 (93.2 % similarity to *N. communis*; possibly a new species within the genus) increased from 0.4 % on day 21 to 1.9 % on day 38, then decreased to 0.04 % on day 78 and to undetectable level thereafter. Relative abundance of OTU-200 (99.1 % similarity to *N. europaea*) increased gradually from undetectable level (prior to day 98) to 0.51 % on day 196, and then declined to undetectable level by day 235. Relative abundance of OTU-16 (95.1 % similarity to *N. europaea*) increased from 0.04 % on day 98 to 3.1 % on day 130, decreased to 1.5 % on day 161, increased to 3.3 % on day 196, and then decreased to 0.8 % on day 206 and to undetectable level thereafter. Relative abundance of OTU-77 (96.6 % similarity to *N. europaea*) increased from 0.05 % on day 98 to 0.7 % on day 196, then decreased to undetectable level thereafter. Relative abundance of OTU-51 (95.7 % similarity to *N. europaea*) increased sharply from undetectable level (prior to day 206) to 6.4 % on day 220, then decreased to 1.0 % on day 235 with little subsequent fluctuation. Relative abundance of OTU-123 (96.1 % similarity to *N. europaea*) increased gradually from 0.02 % on day 220 to 2.0 % on day 290.

### Local blast analysis of nitrifying bacteria (AOB and NOB)

**Fig. 4:**
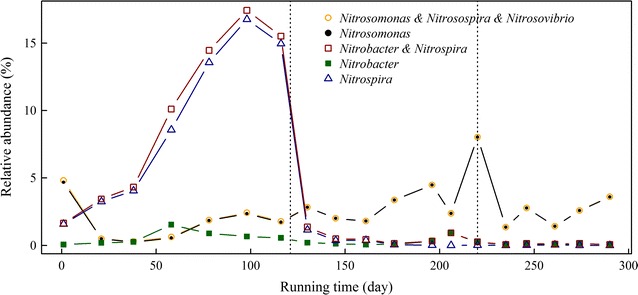
Relative abundance of nitrifying bacteria in SHARON defined from local blast analysis. *Dotted lines* as in Fig. 1

To elucidate relative abundances of autotrophic AOB and NOB taxa, we set up a local database for each sample based on Illumina sequencing data, and obtained a total of 19 local databases for local blast analysis. Trimmed 16S rRNA fragments for all known members of AOB and NOB were used for blast analysis of the local databases using 95 and 97 % similarity cutoffs. Few sequences were defined at 97 % similarity cutoff, but several sequences were found to be affiliated with AOB or NOB at 95 % similarity cutoff. For AOB, in addition to the genus *Nitrosomonas* as described above, the genera *Nitrosospira* and *Nitrosovibrio* were defined with extremely low relative abundance (0.042 and 0.023 %, respectively), detected only during the CFM period. The combined relative abundance of *Nitrosomonas* + *Nitrosospira* + *Nitrosovibrio* was nearly the same as that of *Nitrosomonas* by itself (from ~0.67 % during the CFM period to 8.0 % by day 220, and thereafter decreased to a near-constant ~1.6 %, Fig. 4), indicating that *Nitrosomonas* was the predominant AOB in SHARON. For NOB, two genera were detected: *Nitrobacter* and *Nitrospira*. Relative abundance of *Nitrospira* increased gradually from 1.6 % on day 1 to 16.8 % on day 98, and decreased rapidly to undetectable level after running manner was changed to SBM on day 121. Relative abundance of *Nitrobacter* increased from 0.06 % (day 1) to 1.54 % (day 58) and decreased to undetectable level thereafter. Relative abundance of *Nitrobacter* + *Nitrospira* combined was only marginally (~0.1 %) higher than that of *Nitrospira* by itself, indicating that *Nitrospira* was the predominant NOB during the CFM period.

### Gene abundance

The functional gene *amoA* of AOB encodes the α-subunit of ammonia monooxygenase, which catalyzes the reaction NH_3_ + 2[H] + O_2_ → NH_2_OH + H_2_O (Hollocher et al. 1981; Li et al. 2015). The 491-bp stretch *amoA* gene has strong capacity for fine-scale differentiation of closely related ammonia oxidizers and has been utilized as a functional gene marker for identification of ammonia-oxidizing microorganisms (Rotthauwe et al. 1997; Wang et al. 2012). The abundances of bacterial 16S rRNA gene and AOB *amoA* gene reflect to some degree the relative abundances of bacteria and ammonia-oxidizing organisms (Bai et al. 2012; Gao et al. 2013; Wang et al. 2012).

**Fig. 5:**
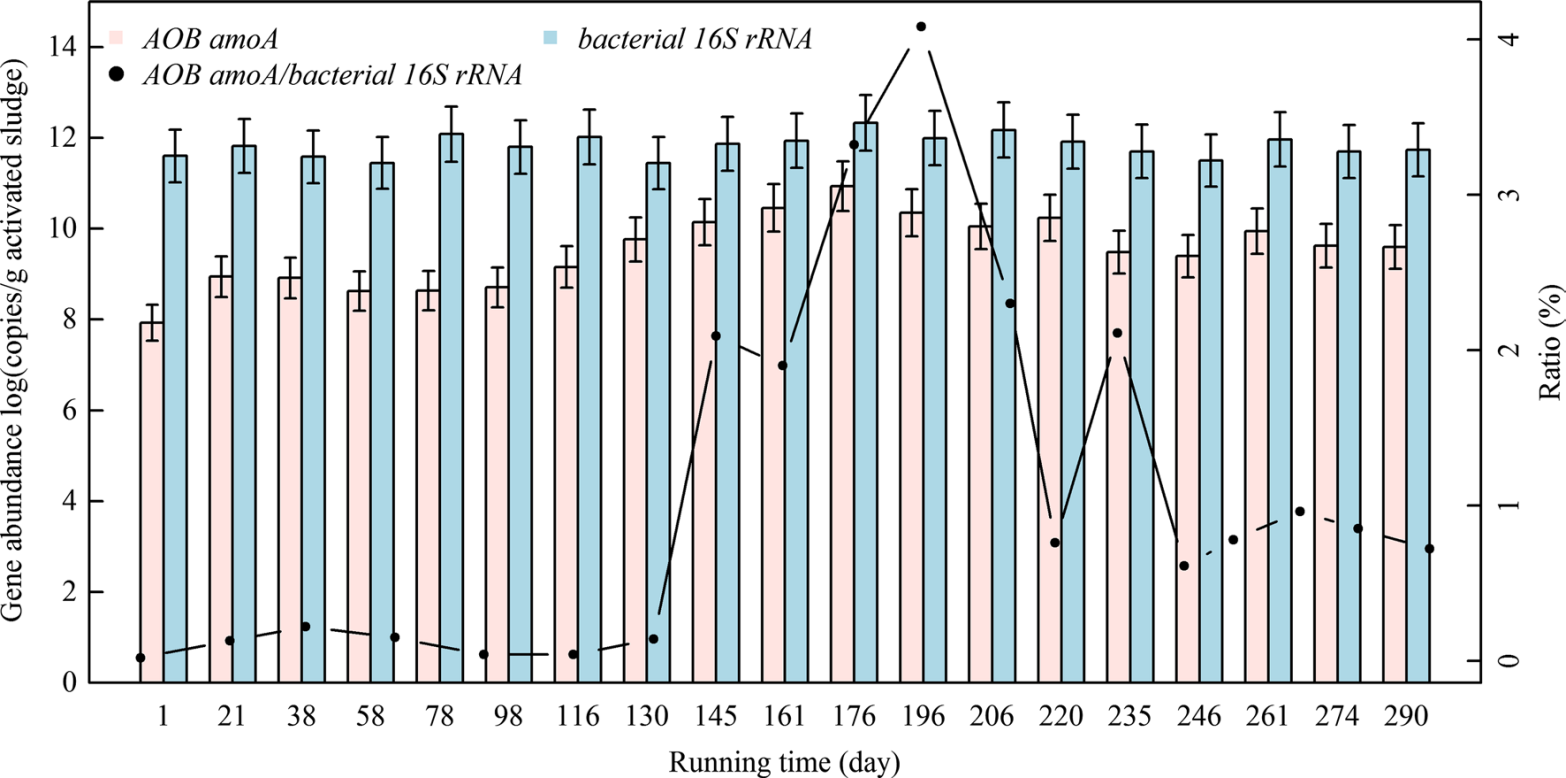
Quantitative analysis of bacterial 16S rRNA and AOB *amoA* genes

We therefore quantified the abundances of bacterial 16S rRNA gene and *amoA* gene by qPCR (Fig. 5). Copy numbers of the two genes were significantly correlated (*R*^*2*^ = 0.58, *p* < 0.001). Throughout the entire experimental period, bacterial 16S rRNA gene copy number ranged from 2.8 × 10^11^ to 2.1 × 10^12^ per g activated sludge, with little fluctuation (Fig. 5). AOB *amoA* gene copy number (per g activated sludge) was 8.5 × 10^7^ on day 1, increased gradually during the CFM period to 1.4 × 10^9^ on day 116, increased further to 5.8 × 10^9^ when running manner was changed to SBM on day 121 and then further increased to 8.7 × 10^10^ by day 176, then gradually decreased to 1.1 × 10^9^ by day 206 (Fig. 5). After the influent was replaced by UASB effluent (day 220), AOB *amoA* gene copy number increased to 1.7 × 10^10^, then decreased to ~4.5 × 10^9^ subsequently, with no effect on partial nitrification performance (Fig. 1).

Bacterial *amoA*/bacterial 16S rRNA gene ratio was <0.2 % during the CFM period, increased to 2.1 % by day 145 (24 days after running manner was changed to SBM), increased further to 4.1 % by day 196, decreased to 0.8 % by day 220, increased again to 2.1 % by day 235, and finally decreased again and remained fairly stable at ~0.78 % (Fig. 5).

### Bacterial *amoA* clone libraries

**Fig. 6:**
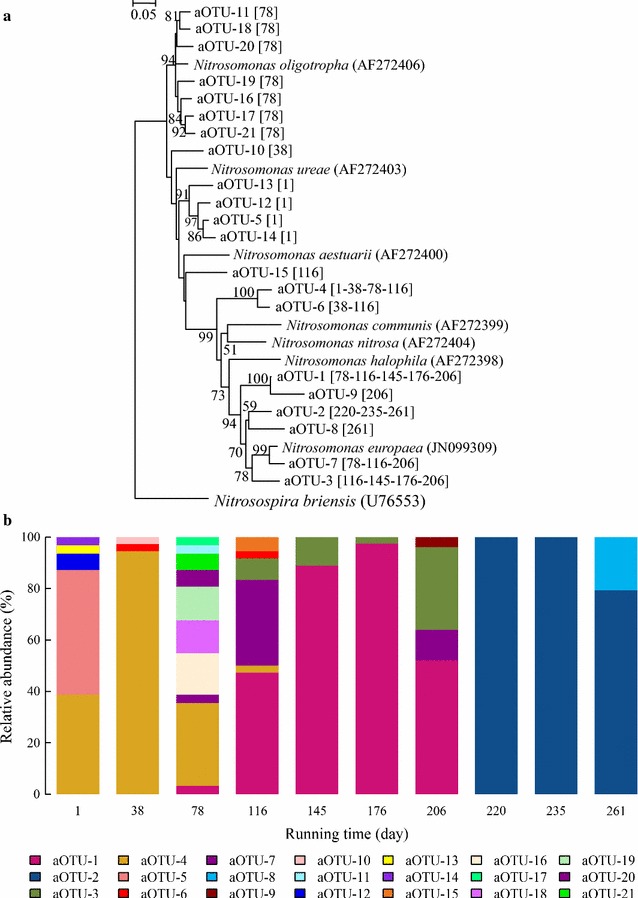
**a** Neighbor-joining tree of aOTUs based on bacterial *amoA* sequences. **b** Relative abundance of these aOTUs. Bootstrap values (>50 %) shown on branch nodes are based on 1000 trials. aOTUs shown in *boldface* are major groups detected in AOB *amoA* gene libraries. Representative sequences obtained are termed “aOTU-”. Numbers in *bracket* following “aOTU-” indicate running time at which samples were obtained. *Bar* evolutionary distance 0.05

Ten AOB *amoA* gene libraries were constructed from the qPCR samples mentioned above. A total of 317 *amoA* gene sequences were obtained, and assigned to 21 aOTUs. Phylogenetic analysis based on 21 representative sequences indicated that these 21 aOTUs could be classified into six groups (Fig. 6a): a *N. oligotropha*-related group (group 1: aOTU-11, aOTU-16, aOTU-17, aOTU-18, aOTU-19, aOTU-20, aOTU-21), an unknown *Nitrosomonas* member-related group (group 2: aOTU-10), a second unknown *Nitrosomonas* member-related group (group 3: aOTU-5, aOTU-12, aOTU-13, aOTU-14), a third unknown *Nitrosomonas* member-related group (group 4: aOTU-15), a fourth unknown *Nitrosomonas* member-related group (group 5: aOTU-4, aOTU-6), and a *N. europaea*-related group (group 6: aOTU-1, aOTU-2, aOTU-3, aOTU-7, aOTU-8, aOTU-9). This phylogenetic tree and that of the AOB OTUs (Fig. 3a) showed very similar topological pattern.

aOTU-4, aOTU-1, and aOTU-2 were respectively predominant during the CFM period, SBM period, and SBM period when UASB effluent was used as influent (Fig. 6b). aOTU-4 was phylogenetically consistent with OTU-99 (Fig. 3a) and had 77.2 % similarity to *N. nitrosa*. Its relative abundance on day 38 was 94.4 % of all *amoA* sequences, and it was the predominant aOTU during days 1–78 (Fig. 6b). aOTU-6, which clustered with aOTU-4, was at or near undetectable level during the entire running period (Fig. 6b). aOTU-1 was phylogenetically consistent with OTU-16 (Fig. 3a) and had 86.9 % similarity to *N. europaea*. Its relative abundance increased gradually from 3.2 % at day 78 to 97.6 % at 176, decreased to 52 % by day 206, and further decreased to undetectable level thereafter (Fig. 6b). aOTU-2 was phylogenetically consistent with OTU-51 (Fig. 3a) and had 86.7 % similarity to *N. europaea*. Its relative abundance was at or near undetectable level during days 1–206, increased to nearly 100 % during days 220–235, then decreased to ~80 % by day 261 (Fig. 6b). Four aOTUs (aOTU-5, aOTU-12, aOTU-13, aOTU-14) were grouped together and detected only on day 1. They were *N. ureae*-related (83.6–86.6 % similarity), and had relative abundance 48.39, 0.06, 0.03, and 0.03 %, respectively (Fig. 6b). They may be phylogenetically consistent with OTU-273 and OTU-1048 (Fig. 3a). Seven aOTUs (aOTU-11, aOTU-16, aOTU-17, aOTU-18, aOTU-19, aOTU-20, aOTU-21) were detected only on day 78, with low relative abundance (0.03–0.16 %). They were clustered with *N. oligotropha* (91.8–94.2 % similarity) (Fig. 6b), and may be phylogenetically consistent with OTU-165, OTU-170, and OTU-1045 (Fig. 3a). aOTU-10 (85.1 % similarity to *N. oligotropha*) was detected only on day 38, with relative abundance 0.03 % (Fig. 6b). aOTU-15 (79.6 % similarity to *N. aestuarii*) was detected only on day 116, with relative abundance 0.06 % (Fig. 6b). The four remaining aOTUs (aOTU-3, aOTU-7, aOTU-8, aOTU-9) were *N. europaea*-related (78.6–94.7 % similarity). aOTU-8 was detected only on day 261, with relative abundance 0.21 %. aOTU-9 was detected only on day 206, with relative abundance 0.04 %. aOTU-7 was detected on days 78, 116, and 206, with relative abundance 3.32, 33.33, and 12 %, respectively. Relative abundance of aOTU-3 increased from 8.33 % on day 116 (the day it was first detected) to 11.11 % on day 145, decreased to 2.44 % by day 176, increased to 32 % by day 206, and declined to undetectable level thereafter (Fig. 6b).

### Bacterial diversity

Good’s coverage estimates of 22 samples were all >99 % (Table 1), indicating that nearly all bacterial species in the reactor were well represented. α-diversity indices were used to assess diversity dynamics of microbial communities in the bioreactor during the entire experimental period (Table 1). The number of OTUs (97 % similarity cutoff) ranged from 185 to 634. Shannon index decreased gradually from 6.45 on day 1 to 2.95 on day 206, and increased further after UASB effluent was used as influent on day 220, up to 6.06 on day 290 (Table 1). All running parameters showed strongly negative correlations with α-diversity calculated based on 16S rRNA genes by Pearson’s test (Additional file 1: Table S3). For α-diversity indices calculated based on AOB *amo*A genes, Good’s coverage estimates were all >93.6 % (except for 87.10 % on day 78), indicating that nearly all AOB in SHARON reactor were included (Table 2). According to Pearson’s test, all running parameters were negatively correlated with α-diversity indices (Additional file 1: Table S4). Running parameters had a positive, but not significant, effect on relative abundance of AOB (Table 3). Relative abundance of NOB had significant negative correlations with numerous running parameters: load ammonia (*R* = −0.65, *p* < 0.01), effluent ammonia (*R* = −0.51, *p* < 0.05), effluent nitrite (*R* = −0.60, *p* < 0.01), DO (*R* = −0.80, *p* < 0.001), and pH (*R* = −0.57, *p* < 0.05) (Table 3).

**Table 1:** OTU richness and diversity indices of microbial communities in the SHARON reactor

Sample^a^	OTU number^b^	Good’s coverage (%)	ACE	Chao I	Shannon	Simpson
CFM-1	617	99.50	719.3	752.8	6.45	0.96
CFM-21	536	99.48	637.5	646.3	5.60	0.93
CFM-38	634	99.34	781.1	779.7	6.33	0.96
CFM-58	599	99.62	651.3	661.2	6.48	0.97
CFM-78	563	99.56	640	651.1	6.49	0.97
CFM-98	569	99.55	646.8	648.5	6.23	0.95
CFM-116	456	99.50	567.6	581.5	5.39	0.93
SBM-130	461	99.46	587	578.7	5.03	0.90
SBM-145	374	99.61	460.1	465	5.72	0.96
SBM-161	299	99.57	424.4	435.5	3.86	0.79
SBM-176	213	99.69	314.6	292.6	3.71	0.83
SBM-196	185	99.77	247.8	247.2	4.17	0.88
SBM-206	155	99.80	210	215.1	2.95	0.69
SBM-220	452	99.62	516.7	554.7	5.58	0.94
SBM-235	455	99.61	522.3	552.5	5.69	0.93
SBM-246	437	99.62	502.8	494.6	4.96	0.87
SBM-261	410	99.62	481	501	4.91	0.86
SBM-274	465	99.62	527.3	550.2	5.58	0.93
SBM-290	456	99.72	491.4	503.3	6.06	0.96
UASB-288	455	99.82	491.7	508.3	5.38	0.93
UASB-304	457	99.77	509.2	511.2	5.30	0.92
UASB-334	488	99.79	529.4	542.6	5.91	0.93

^a^Numbers following “CFM”, “SBM”, and “UASB” in this column indicate sampling date

^b^97 % similarity cutoff

**Table 2:** aOTU richness and diversity indices of AOB communities based on *amoA* genes in this SHARON

Sample^a^	Clone number	aOTU number^b^	Good’s coverage (%)	Chao I	Shannon
CFM-1	38	5	93.6	5.5	1.61
CFM-38	36	2	94.44	4.0	0.36
CFM-78	32	10	87.10	12	2.86
CFM-116	37	6	94.44	6.5	1.86
SBM-145	27	2	100	2	0.50
SBM-176	41	2	97.56	2	0.17
SBM-206	26	4	96	4	1.57
SBM-220	29	1	100	1	0
SBM-235	22	1	100	1	0
SBM-261	29	2	100	2	0.74

^a^Numbers following “CFM” and “SBM” in this column indicate sampling date

^b^97 % similarity cutoff

**Table 3:** Correlations (*R* values) between AOB/NOB and running parameters from Pearson’s test

Bacteria	NH_4_^+^-N-influent	NH_4_^+^-N-effluent	NO_2_^−^-N-effluent	DO	pH
AOB	0.30	0.17	0.43	0.36	0.33
NOB	−0.65**	−0.51*	−0.60**	−0.80***	−0.57*

* *p* < 0.05, ** *p* < 0.01, *** *p* < 0.001

**Fig. 7:**
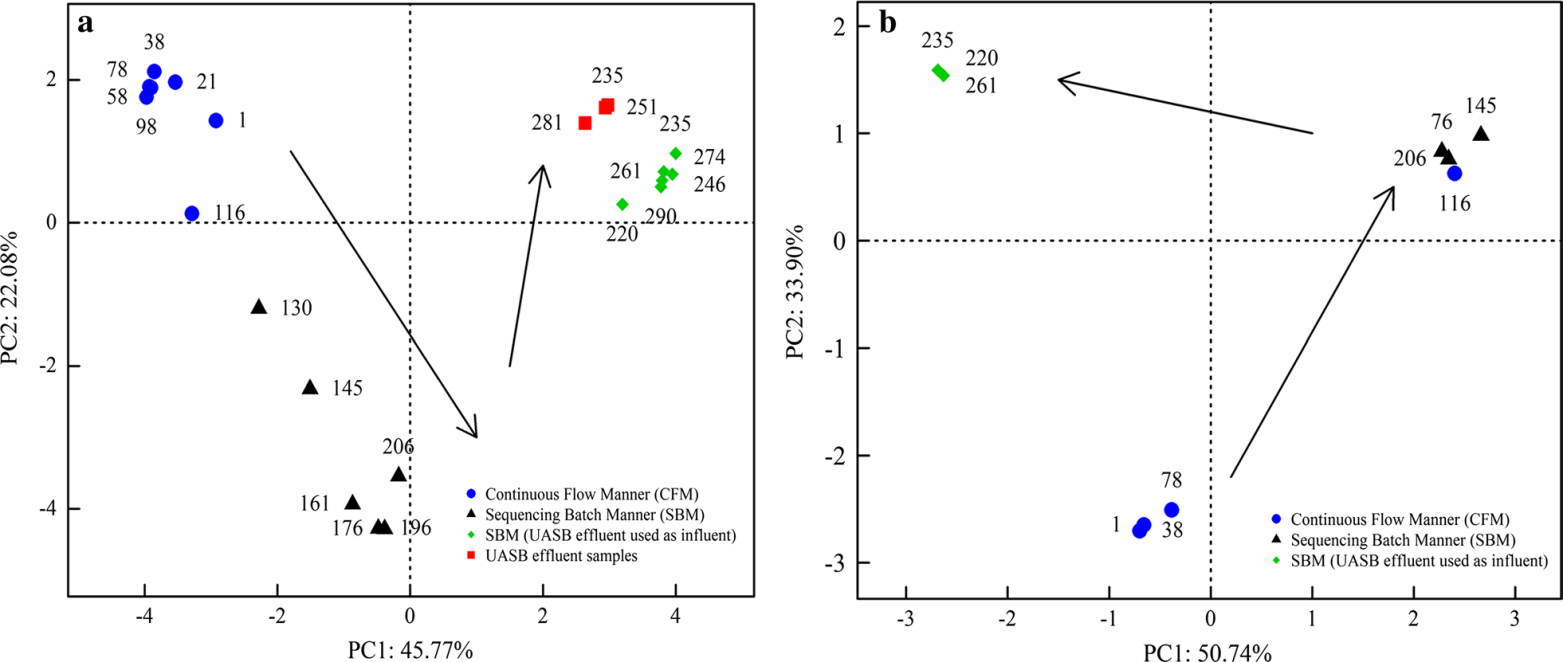
Principal coordinate analysis (PCoA) based on Bray–Curtis distance, from bacterial 16S rRNA gene sequences (**a**) and AOB *amoA* gene sequences (**b**)

Application of PCoA resulted in clustering of 19 SHARON samples into three groups (days 1–116, days 130–196, days 220–290) related to the time points when running manner was changed from CFM to SBM, and to SBM with UASB effluent used as influent (Fig. 7a). Samples from days 220 to 290 clustered with UASB effluent samples when UASB effluent was used as influent for SHARON on day 220 (Fig. 7a). ANOSIM (analysis of similarity) revealed significant differences between the three groups (*R*^*2*^ = 0.94, *p* < 0.01). RDA (redundancy analysis) revealed the dependence of community dynamics on environmental factors (high load ammonia, effluent ammonia, effluent nitrite, pH, DO) during the entire running period (Additional file 1: Figure S3). Pearson’s test showed that running parameters had significant effects on relative abundance of the major phyla (Table 4). PCoA based on 317 AOB *amoA* sequences showed nearly the same dynamic pattern as PCoA based on 16S rRNA sequences (Fig. 7b). Samples obtained during the SBM period were separated from those obtained during the CFM period, except for the day 116 sample (Fig. 7b). Samples obtained from day 220 to 261 (when UASB effluent was used as influent) were separated from those obtained with artificial wastewater used as influent (Fig. 7b).

**Table 4:** Correlations (*R* values) between major phyla and running parameters from Pearson’s test

phylum	influent NH_4_^+^-N	effluent NH_4_^+^-N	effluent NO_2_^−^-N	DO	pH
*Bacteroidetes*	0.55*	0.23	0.86***	0.65**	0.64**
*Proteobacteria*	−0.60**	−0.32	−0.86***	−0.70***	−0.78***
*Nitrospirae*	-0.69**	−0.53**	−0.62**	−0.83***	−0.60**
*TM7*	0.4	0.53*	0.06	0.29	0.60**
*Chloroflexi*	-0.50*	−0.54*	−0.19	−0.33	−0.48*
*Chlorobi*	−0.09	−0.14	−0.27	−0.17	−0.38
*Firmicutes*	0.45	0.46*	0.15	0.56*	−0.09
*Actinobacteria*	0.23	−0.05	0.70***	0.49*	0.32
*Synergistetes*	0.17	−0.04	0.46*	0.39	0.18
*Gemmatimonadetes*	−0.35	−0.56**	0.18	−0.05	−0.22
*Planctomycetes*	−0.79***	−0.63**	-0.70***	−0.86***	−0.68***

* *p* < 0.05, ** *p* < 0.01, *** *p* < 0.001

## Discussion

Most studies to date on bacterial communities and AOB in nitrifying bioreactors have focused on activated sludge samples obtained from a single time point (Gao et al. 2013; Limpiyakorn et al. 2006; Wells et al. 2009; Zhang et al. 2011), which does not reflect bacterial community succession during the startup period or entire running period, and does not allow determination of the environmental factors that drive community dynamics. A novel treatment system termed “UASB + SHARON + ANAMMOX” was constructed in our laboratory for treatment of piggery wastewater with high contents of COD and ammonia. The SHARON reactor was expected to achieve 50 % short-term nitrification and ensure effluent NH_4_^+^-N/NO_2_^−^-N ratio ~1. In the present study, we investigated dynamics of bacterial community diversity, structure, and composition during the entire running period, including startup, domestication, and replacement of artificial wastewater with real wastewater. We analyzed the predominant functional bacterial taxa during different running periods, and the effects of various environmental and engineering factors on reactor performance.

### Bacterial community dynamics

In this SHARON reactor, bacterial diversity showed a general decreasing trend during days 1–206, and increased slightly following day 220, when UASB effluent was used as influent (Table 1). Pearson’s test showed that our running parameters were negatively correlated with α-diversity of the microbial community (Additional file 1: Table S3). The decrease in bacterial diversity may have resulted from domestication of artificial wastewater with high ammonia content, similarly to the observations of Whittenbury et al. (1970). The slight increase in diversity after UASB effluent was used as influent may have resulted from the introduction of groups such as *Spirochaetae*, *Synergistetes*, *Thermotogae*, *WWE1*, and *WS6* present in UASB effluent (Fig. 2a). Entire microbial community dynamics and AOB community dynamics displayed very similar succession trends, according to PCoA (Fig. 7). Entire microbial community dynamics underwent two major changes associated with change of running manner and use of UASB effluent as influent, and bacterial communities were clustered into three groups (Fig. 7a) having significantly different compositions (*R*^*2*^ = 0.94; *p* < 0.01). This observation was not surprising, because change of running manner has been shown to greatly alter community composition for adaptation to environmental changes (Turner et al. 1998; Wells et al. 2009). In PCoA based on AOB *amoA* genes, the day 116 sample was separated from the CFM samples and clustered with SBM samples (Fig. 7b). This observation suggests that evolutionary trends of the AOB community differed from those of the entire microbial community, consistently with the findings of Zhang et al. (2011).

*Bacteroidetes* and *Proteobacteria* were the primary bacterial phyla found in this SHARON, as they generally are in activated sludge (Juretschko et al. 1998, 2002; Kong et al. 2002; Xia et al. 2008). The relative abundances of these two phyla were inversely correlated (Table 4), presumably because of changes in running parameters; high DO levels and pH values favor growth of *Bacteroidetes* (Gao et al. 2011; Hu et al. 2015). The low relative abundance of *Planctomycetes* during the CFM period and its further decline to undetectable level after change of running manner to SBM (Fig. 2a) may have been due to the increase of DO level from 0.7–1.5 to 7.0–8.0 mg/L) (*R* = −0.86, *p* < 0.001), because these bacteria are anaerobic ammonia oxidizers (Innerebner et al. 2007; Strous et al. 1999). The gradual increase of relative abundance of *Nitrospirae*, a NOB (Daims et al. 2015), during the CFM period, with little nitrate accumulation in effluent (Figs. 1, 2a), and its decrease following change to SBM, may have been related to inhibitory effects of high pH (~10; *R* = −0.57, *p* < 0.01) and high ammonium concentration (600–900 mg/L; *R* = −0.65, *p* < 0.01) in influent. High pH and load ammonia were previously reported to be the major factors that inhibit NOB growth (Balmelle et al. 1992; Bernet et al. 2001; Garrido et al. 1997). The phylum *TM7* showed high relative abundance only during days 161–196 (Fig. 2a), perhaps because of high values of both load and effluent ammonia, which are positively correlated with *TM7* abundance (Table 4). The mechanisms underlying such short-term high abundance, and its effects in this SHARON, await further investigation.

### Dynamics of ammonia oxidizers, nitrite oxidizers, and nitrite accumulation

Ammonia can be oxidized to nitrite by both AOB and ammonia-oxidizing archaea (AOA). AOB populations (Fig. 3a) and AOB *amoA* genes (Figs. 5, 6) were detected in the present study, but AOA populations and AOA *amoA* genes were not. AOB is much more abundant than AOA in activated sludge (Mussmann et al. 2011; Wang et al. 2012; Zhang et al. 2009). AOA are typically found in habitats with low ammonia concentration, low DO level, and/or acidic conditions (low pH) (Gao et al. 2013; Zhang et al. 2012). They were barely detectable in this SHARON, which had high DO level and ammonia content. Pearson’s test showed that the high DO and ammonia also greatly affected α-diversity of AOB (Additional file 1: Table S4). These findings are consistent with those of Lydmark et al. (2007) and Wang et al. (2012).

The real relative abundance of AOB in this SHARON may be much higher than our estimate based on 16S rRNA gene sequencing through Illumina MiSeq method (Figs. 3 and 4). The average number of 16S rRNA operons in heterotrophic bacteria has been estimated as 5.5 in nutritional environments (Klappenbach et al. 2000, 2001; Nadkarni et al. 2002), and there is only one copy of 16S rRNA in AOB (Aakra et al. 1999). Surprisingly, the AOB *amoA* gene/bacterial 16S rRNA gene ratio calculated based on qPCR was less than the relative abundance of AOB estimated based on 16S rRNA genes. There are 2–3 *amoA* copies in every AOB cell for β-subdivision of *Proteobacteria* (Norton et al. 2002; Okano et al. 2004). The difference may be due to biases arising from the quantitative process, since the AOB *amoA* primers used were designed based on *N. europaea* (He et al. 2007; Rotthauwe et al. 1997).

The topologies of phylogenetic trees based on OTUs and aOTUs were similar, and indicate that *Nitrosomonas* was the predominant genus in this SHARON. Groups generated from the two trees were generally congruent but not identical (Figs. 3a, 6b), because of the inconsistency in analysis of AOB through 16S rRNA and *amoA* genes (Purkhold et al. 2003). Many AOB OTUs and aOTUs showed <97 % sequence similarity to known AOB species, suggesting that many novel AOB taxa were present in this SHARON.

Average relative abundance of AOB was ~1 % during CFM with no nitrite accumulation during days 1–120, with low DO (0.7–1.5 mg/L), low influent ammonia concentration (NH_4_^+^-N, 100–200 mg/L), and no pH control (Fig. 1), demonstrating that these running parameters were not favorable for AOB. The predominant AOB during this CFM period were unidentified ones represented by OTU-99/aOTU-4 and the *N. oligotropha*-related group (OTU-165/170/1045; aOTU-11/16-21), which were able to adapt to the acidic environment (Gieseke et al. 2006). The presence of *Nitrospira* may have led to the failure of nitrite accumulation despite the presence of *N. oligotropha*-related AOB. When running manner was changed to SBM, high influent ammonia concentration (NH_4_^+^-N, 600–900 mg/L) with high pH (~10) and high DO level (7.0–8.0 mg/L) increased the relative abundance of AOB, and the *N. oligotropha*-related group was replaced by the *N. europaea*-related group, with nitrite accumulation in the effluent (Figs. 1, 3, 5). These changes may have been due to altered parameters resulting from the change of running manner. AOB activity is inhibited under acidic conditions because the bioavailability of ammonia is reduced by ionization (Downing and Nere 1964; Gerardi 2003; Suzuki et al. 1974), and high pH increases the concentration of molecular ammonia. The Ks value of NH_3_ oxidation is much lower for *N. oligotropha* (2.4–4.2 µM) than for *N. europaea* (30–56 µM) (Koops et al. 1991; Koops and Pommerening-Röser 2005). *Nitrosomonas europaea* has higher affinity for oxygen than does *N. oligotropha* (Park and Noguera 2007).

Ammonia-oxidizing activity was enhanced when UASB effluent was used as influent, despite the reduced relative abundance of AOB (Fig. 4). One possible explanation is that alkaline organic materials in the UASB effluent maintained an alkaline pH during the entire SBR batch. AOB activity may also have been promoted by organic compounds (e.g., formate, acetate, pyruvate, glucose, peptone) present in the UASB effluent (Krummel and Harms 1982).

The families *Rhodocyclaceae, Comamonadaceae*, *Xanthomonadaceae, Pseudomonadaceae*, and *Sphingomonadaceae* were detected at moderate levels in this SHARON (Fig. 2b). Certain members of these families have been identified as heterotrophic AOB (Bal Krishna et al. 2013; Huang et al. 2015; Khardenavis et al. 2007; Kim et al. 2008), and may have contributed to nitrite accumulation in this SHARON. This possibility requires further investigation.

In conclusion, the novel “UASB + SHARON + ANAMMOX” system described here utilized SBM for partial nitrification, following CFM, to transform 50 % of ammonia to nitrite. The pattern of microbial community dynamics was nearly the same as that of AOB community dynamics. *Nitrosomonas europaea*-related bacteria were the autotrophic AOB primarily responsible for partial nitrification during SBM. Ammonia-oxidizing activity was enhanced by the high DO level, high ammonia (NH_4_^+^-N) concentration, high pH, and presence of organic materials in UASB effluent used as influent.

## Additional file

10.1186/s13568-016-0245-5 Additional figures and tables.

## Abbreviations

UASB: upflow anaerobic sludge blanket
SHARON: single reactor system for high activity ammonium removal over nitrite
anammox/ANAMMOX: ANaerobic AMMonium OXidation
AOB: ammonium oxidation bacteria
AOA: ammonium oxidation archaea
NOB: nitrite oxidation bacteria
CFM: continuous flow manner
SBM: sequencing batch manner
HRT: hydraulic retention time
DO: dissolved oxygen
COD: chemical oxygen demand
QIIME: quantitative insights into microbial ecology
qPCR: quantitative polymerase chain reaction
OTU: operational taxonomic units
PCoA: principal coordinates analysis
RDA: redundancy analysis
ANOSIM: analysis of similarity
NJ: neighbor-joining
