# Supplementary material for: Association of running manner with bacterial community dynamics in a partial short-term nitrifying bioreactor for treatment of piggery wastewater with high ammonia content
Source: AMB Express. 2016 Sep 15;6:76. doi: 10.1186/s13568-016-0245-5 (PMC5025419; doi:10.1186/s13568-016-0245-5)

# AMB Express

# Supplementary information for:

**Association of running manner with bacterial community dynamics in a partial short-term nitrifying bioreactor for treatment of piggery wastewater with high ammonia content**

Wei-Li Du^1, 2^ (duweili789@163.com), Qiang Huang^1, 2^ (15250967477@163.com), Li-Li Miao^1^ (miaoll@im.ac.cn), Ying Liu^1^ (liuying@im.ac.cn), Zhi-Pei Liu^1*^ (liuzhp@im.ac.cn)

^1^ State Key Laboratory of Microbial Resources, Institute of Microbiology, Chinese Academy of Sciences, Beijing 100101, P. R. China. ^2^ University of Chinese Academy of Sciences, Beijing 100049, P.R. China.

* Correspondence: Zhi-Pei Liu, Institute of Microbiology, Chinese Academy of Sciences, No. 1 West Beichen Road, Chaoyang District, Beijing 100101, P. R. China, Tel: +10-64806081

## Supplementary Methods

Analytical methods of nitrogen compound, COD, pH, DO

NH_4_^+^-N: Nessler assay .

NO_2_^-^-N: N-(1-naphthyl)-1, 2-diaminoethane dihydrochloride spectrophotometry .

NO_3_^-^-N: thymol spectrophotometry .

COD: using a COD instrument (model CTL-12; Chengde Huatong Environmental Protection Equipment Co., Chengde, China).

pH: measured with a pH meter (model PB-10; Sartorius, Germany).

DO: determined with a DO meter (model JPSJ-605; Shanghai Precision & Scientific Instrument Co., Shanghai, China).

**References**

Liu SX, Li B (2006) The application of thymol crystals spectrophotometry method. Modern Medicine Journal of China 8:4

Mahmood Q, Zheng P, Hayat Y, Jin RC, Azim MR, Jilani G, Islam E, Ahmed M (2009) Effect of nitrite to sulfide ratios on the performance of anoxic sulfide oxidizing reactor. Arab J Sci Eng 34(1A):45-54

Zhang Q (2009) Research on key issues in determination of ammonia nitrogen in water and wastewater by Nessler's reagent spectrophotometry. Environment Engineering 27:85

**Legends to Supplementary Figures**

**Supplementary Figure S1**. Schematic diagram of the “UASB+SHARON+ANAMMOX” system for piggery wastewater treatment. (1) storage tank; (2) delivery pump; (3) gas meter; (4) UASB reactor; (5) effluent tank; (6) delivery pump; (7) warm water delivery pump; (8) SHARON reactor; (9) thermostat water bath; (10) air pump; (11) effluent tank; (12) suction pump; (13) delivery pump; (14) ANAMMOX reactor; (15) warm water delivery pump; (16) effluent tank.

**Supplementary Figure S2**. Heatmap plot illustrating relative percentages of major genera (clustering shown on vertical axis) within each sample (horizon-axis clustering). Numbers at bottom: times when activated sludge samples were obtained. Color intensities indicate relative abundances at genus level (legend at bottom).

**Supplementary Figure S3**. Effects of environmental variables on bacterial community dynamics, from redundancy analysis (RDA) based on Illumina MiSeq data. Numbers: sampling dates. Arrows: direction and magnitude of environmental variables that drove bacterial community dynamics. Environmental variables were selected based on significance (*p*< 0.05) calculated from Mantel test results.

**Supplementary Tables**

**Table S1**. Parameters of component reactors of “UASB+SHARON+ANAMMOX” system for piggery wastewater treatment.

| Reactor | UASB | SHARON | ANAMMOX |
| --- | --- | --- | --- |
| Height/diameter  Effective volume (L)  Temperature (ºC)  Hydraulic retention time (h) | 12/1  13.3  31-32  26.6 | 10/3  12.5  room temperature  25 | 12/1  13.3  31-32  26.6 |

**Table S2**. Influent characteristics of SHARON reactor at various stages.

| Running time (day) | Stage | NH_4_^+^-N (mg/L) | Characteristics | pH |
| --- | --- | --- | --- | --- |
| 1-65  66-120  121-127  128-142  143-162  163-177  178-196  197-202  203-219  220-290 | CFM  CFM  SBM  SBM  SBM  SBM  SBM  SBM  SBM  SBM | ~100  ~200  200→300  300→600  600→900  900→1000  1000→800  800→600  ~600  ~600 | AW  AW  AW  AW  AW  AW  AW  AW  AW  EPW | ~7.5  ~7.5  ~8  ~8  ~10  ~10  ~10  ~10  ~10  8.2-8.6 |

AW: Artificial wastewater; ingredients (w/w): NH_4_Cl: glucose: NaHCO_3_: Na_2_HPO_4_ (16:4:2:1).

EPW: Effluent from UASB treatment of real piggery wastewater.

CFM: Continuous flow manner.

SBM: Sequencing batch manner.

**Table S3**. Correlations (*R* values) between α-diversity indices of microbial communities and running parameters by Pearson's test.

| α-diversity | NH_4_^+^-N-influent | NH_4_^+^-N-effluent | NO_2_^-^-N-effluent | DO | pH |
| --- | --- | --- | --- | --- | --- |
| ACE  Chao 1  Shannon  Simpson | -0.71***  -0.71***  -0.64**  -0.50* | -0.79***  -0.80***  -0.69**  -0.53* | -0.56*  -0.52*  -0.49*  -0.46* | -0.74***  -0.72***  -0.62**  -0.51* | -0.79***  -0.81***  -0.79***  -0.65** |

* *p*<0.05, ** *p*<0.01, *** *p*<0.001.

**Table S4**. Correlations (*R* values) between α-diversity indices of AOB communities and running parameters by Pearson's test.

| α-diversity | NH_4_^+^-N-influent | NH_4_^+^-N-effluent | NO_2_^-^-N-effluent | DO | pH |
| --- | --- | --- | --- | --- | --- |
| Chao 1  Shannon | -0.46  -026 | -0.54  -0.36 | -0.65*  -0.46 | -0.85**  -0.68* | -0.55  -0.48 |

* *p*<0.05, ** *p*<0.01.

**Supplementary Figure S1**


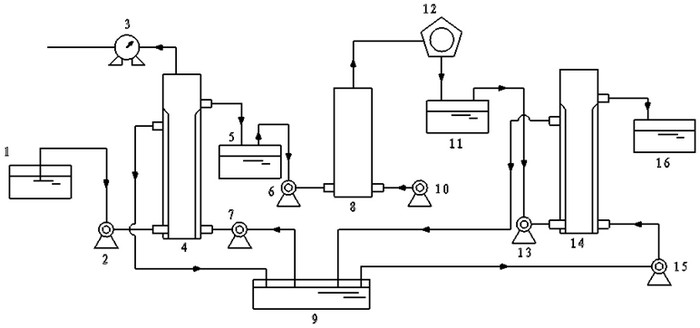


**Supplementary Figure S2**





**Supplementary Figure S3**


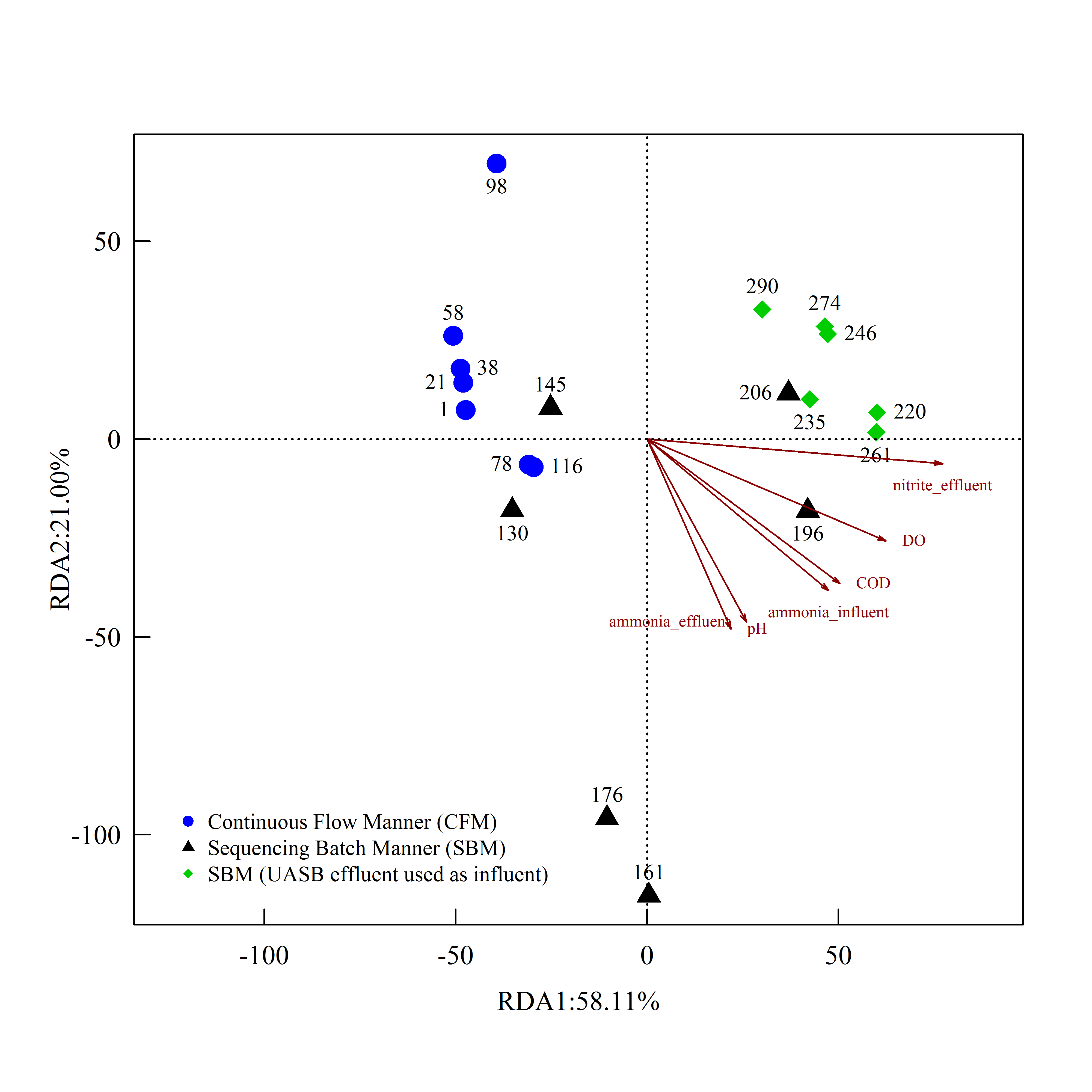

Supplement: Supplementary file 1 — 10.1186/s13568-016-0245-5 Additional figures and tables. [file 13568_2016_245_MOESM1_ESM.doc]
